# Improving sexual health in men with prostate cancer: randomised controlled trial of exercise and psychosexual therapies

**DOI:** 10.1186/1471-2407-14-199

**Published:** 2014-03-18

**Authors:** Prue Cormie, Suzanne K Chambers, Robert U Newton, Robert A Gardiner, Nigel Spry, Dennis R Taaffe, David Joseph, M Akhlil Hamid, Peter Chong, David Hughes, Kyra Hamilton, Daniel A Galvão

**Affiliations:** 1 Edith Cowan University Health and Wellness Institute, Edith Cowan University, 270 Joondalup Drive, Joondalup, WA 6027, Australia; 2 Griffith Health Institute, Griffith University, Southport, Australia; 3 Cancer Council Queensland, Brisbane, Australia; 4 Prostate Cancer Foundation of Australia, Sydney, Australia; 5 Department of Urology, Royal Brisbane and Women’s Hospital, Brisbane, Australia; 6 Centre for Clinical Research, The University of Queensland, Brisbane, Australia; 7 Department of Radiation Oncology, Sir Charles Gairdner Hospital, Nedlands, Australia; 8 Faculty of Medicine, University of Western Australia, Nedlands, Australia; 9 School of Environmental and Life Sciences, University of Newcastle, Ourimbah, Australia; 10 Department of Urology, Royal Perth Hospital, Perth, Western Australia, Australia; 11 Lake Macquarie Urology, Newcastle, New South Wales, Australia; 12 Bangalow Community Health Centre, Bangalow, New South Wales, Australia

**Keywords:** Prostate cancer, Sexual health, Erectile dysfunction, Exercise, Resistance training, Aerobic exercise, Psychosexual support, Self-management

## Abstract

**Background:**

Despite being a critical survivorship care issue, there is a clear gap in current knowledge of the optimal treatment of sexual dysfunction in men with prostate cancer. There is sound theoretical rationale and emerging evidence that exercise may be an innovative therapy to counteract sexual dysfunction in men with prostate cancer. Furthermore, despite the multidimensional aetiology of sexual dysfunction, there is a paucity of research investigating the efficacy of integrated treatment models. Therefore, the purpose of this study is to: 1) examine the efficacy of exercise as a therapy to aid in the management of sexual dysfunction in men with prostate cancer; 2) determine if combining exercise and brief psychosexual intervention results in more pronounced improvements in sexual health; and 3) assess if any benefit of exercise and psychosexual intervention on sexual dysfunction is sustained long term.

**Methods/Design:**

A three-arm, multi-site randomised controlled trial involving 240 prostate cancer survivors will be implemented. Participants will be randomised to: 1) ‘Exercise’ intervention; 2) ‘Exercise + Psychosexual’ intervention; or 3) ‘Usual Care’. The Exercise group will receive a 6-month, group based, supervised resistance and aerobic exercise intervention. The Exercise + Psychosexual group will receive the same exercise intervention plus a brief psychosexual self-management intervention that addresses psychological and sexual well-being. The Usual Care group will maintain standard care for 6 months. Measurements for primary and secondary endpoints will take place at baseline, 6 months (post-intervention) and 1 year follow-up. The primary endpoint is sexual health and secondary endpoints include key factors associated with sexual health in men with prostate cancer.

**Discussion:**

Sexual dysfunction is one of the most prevalent and distressing consequences of prostate cancer. Despite this, very little is known about the management of sexual dysfunction and current health care services do not adequately meet sexual health needs of survivors. This project will examine the potential role of exercise in the management of sexual dysfunction and evaluate a potential best-practice management approach by integrating pharmacological, physiological and psychological treatment modalities to address the complex and multifaceted aetiology of sexual dysfunction following cancer.

**Trial registration:**

Australian New Zealand Clinical Trials Registry ACTRN12613001179729.

## Background

Increasing prostate cancer incidence (~56% increase since 1991) and survival rates (5-year survival rate increased from ~58% to ~92% since 1987) coupled with an aging population have led to a large and rapidly growing population with unique health care requirements [1]. Sexual dysfunction is one of the most common, distressing and persistent adverse effects of prostate cancer treatments [2-11] which has a profound impact on quality of life both for the patient and his partner [2-4,10,12-14]. The level of concern associated with sexual dysfunction is reflected by the willingness of men to sacrifice survival for sexual potency (i.e. 68% of men are willing to sacrifice a ~10% greater advantage in 5-year survival to maintain sexual function) [15]. Up to 90% of men will experience sexual dysfunction following primary therapy for prostate cancer with treatments frequently leading to erectile dysfunction, loss of libido, penile shortening and altered orgasmic experience [2-11]. Current health care services are inadequate to address the demand for management of sexual dysfunction [3], with 47% of prostate cancer survivors reporting unmet sexual health care needs [16]. Management strategies predominately involve pharmacological interventions to address the direct physiological effects of prostate cancer treatment on erectile function [17,18]. However, the aetiology of sexual dysfunction is multifaceted and there are considerable physiological and psychological side effects of prostate cancer treatments which contribute to sexual dysfunction that are not counteracted by pharmacological intervention [3,4,10]. Exercise has established efficacy for improving many of these factors in prostate cancer patients including changes in body composition (especially to counteract body feminisation with androgen deprivation therapy [ADT]), fatigue, physical function, risk of co-morbid conditions, inflammatory state, depression, anxiety and quality of life [19-25]. Emerging data indicates that exercise also fosters improved feelings of masculinity and has a positive impact on libido in men with prostate cancer [26,27], a concern that is highly prevalent and difficult to treat [3,12]. Furthermore, psychological therapies have established efficacy for improving treatment induced psychological changes associated with prostate cancer including depression and anxiety as well as enhanced quality of life [28-31] with emerging evidence for improving sexual health in prostate cancer patients [32,33]. Therefore, a multidisciplinary management strategy incorporating pharmacological (usual medical care), physiological (exercise program) and psychological (brief psychosexual self-management) interventions may represent a best-practice model for addressing sexual dysfunction secondary to prostate cancer treatment [27]. The relatively low uptake, compliance and satisfaction with current treatment options [34-36] coupled with the low help-seeking and health service utilisation behavior of men [37-39] provides additional rationale for the novel management approach proposed. Hence, the aims of this study are to:

1. Examine the efficacy of exercise as a therapy to aid in the management of sexual dysfunction in men with prostate cancer.

2. Determine if combining exercise and brief psychosexual self-management results in more pronounced improvements in the sexual health of men with prostate cancer.

3. Assess if any benefit of exercise and brief psychosexual self-management on sexual dysfunction in men with prostate cancer is sustained long term.

We will evaluate three main hypotheses: 1) Compared with usual medical care, exercise will improve sexual health in men with prostate cancer who are concerned by sexual dysfunction. We theorise that exercise will improve masculine self-esteem, quality of life, psychological distress, fatigue, body composition, body image and physical function, culminating in increased sexual health; 2) When exercise and brief psychosexual self-management are combined, improvements in sexual health will exceed those observed in usual medical care and exercise therapy alone. We theorise that brief psychosexual self-management will further enhance improvements in sexual health through increasing men’s ability to better self-manage their well-being and sexual dysfunction (i.e. enhanced uptake of pharmacologic management of erectile dysfunction); and 3) Improvements in sexual health will be sustained 1 year after completion of the exercise and combined exercise and psychosexual interventions. We hypothesise that the theoretically based interventions will prompt behavioural change that leads to sustained improvements in sexual health.

Despite being a critical survivorship care issue, there is a clear gap in current knowledge of the optimal treatment of sexual dysfunction in men with prostate cancer. The current study will generate information to address this gap. There is a strong theoretical rationale [27] and emerging evidence [26] that exercise is an innovative therapy to counteract sexual dysfunction in men with prostate cancer. However, there is a distinct lack of research investigating the efficacy of exercise on sexual health following cancer treatment. Furthermore, despite the multidimensional aetiology of sexual dysfunction, there is a paucity of research investigating the efficacy of integrated treatment models. This study will address these limitations. Findings will expand current clinical guidelines for the management of sexual dysfunction in men with prostate cancer and, importantly, facilitate the development of targeted supportive care services for survivors concerned by their sexual health. Evidence gained may lead to a paradigm shift in the management of sexual dysfunction in prostate cancer survivors.

## Methods/Design

**Figure 1:**
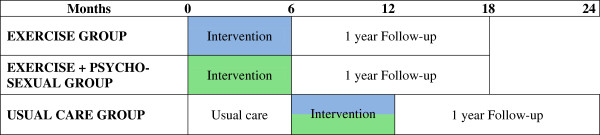
**Summary of the study design.** Self-report measures also assessed at 6 months follow-up (i.e. at 12 months for both the Exercise and the Exercise + Psychosexual groups; 18 months for the usual care group).

A single-blinded (investigators blinded to group allocation), three arm, multi-site randomised controlled trial (RCT) design will be used to examine the efficacy of exercise and psychosexual therapies on sexual health in men with prostate cancer. An ‘Exercise’ group will complete the exercise intervention, an ‘Exercise + Psychosexual’ group will complete the same exercise intervention as well as a brief psychosexual self-management intervention and a ‘Usual Care’ group will maintain usual medical care for a period of 6 months. The Usual Care group will be offered participation in the interventions at the completion of the 6-month period (half to receive the Exercise intervention and half the Exercise + Psychosexual intervention). The RCT will be followed by a prospective cohort study examining the long-term impact of exercise versus exercise and brief psychosexual self-management on sexual dysfunction in prostate cancer patients 1 year after the interventions (Figure 1). The study will be guided by the CONSORT statement [40].

### Participants

**Figure 2:**
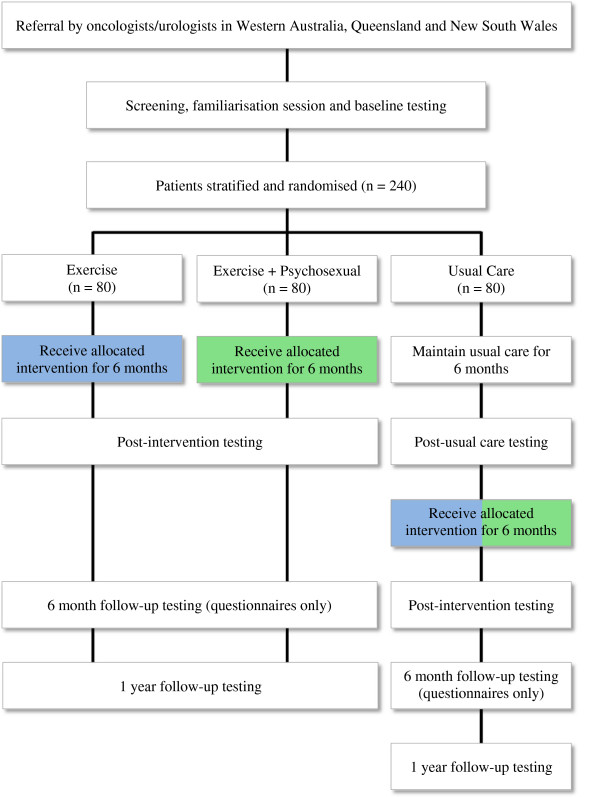
CONSORT diagram depicting the flow of participants throughout the trial.

Two hundred and forty men (80 subjects per arm) treated for prostate cancer will be recruited by invitation from their attending specialist (oncologist/urologist). Participants will be recruited in Perth, Western Australia; Brisbane, Queensland; Central and North Coasts of New South Wales. Inclusion criteria are: 1) concern about sexual health as assessed by an International Index of Erectile Functioning (IIEF) overall satisfaction score < 8 (i.e. moderately-very dissatisfied) [41] and/or Expanded Prostate Cancer Index Composite (EPIC) sexual bother score > 8 (i.e. small-big problem) [42]; 2) prior/current treatment for prostate cancer including prostatectomy, radiotherapy or ADT; and 3) physician consent. Exclusion criteria are: 1) non-nerve sparing prostatectomy; 2) > 6 months since prostatectomy or completion of radiotherapy or ADT; 3) incontinence defined as requiring the use of > 1 pad in a 24-hour period; 4) already performing regular exercise defined as undertaking structured aerobic or resistance training two or more times per week within the past 3 months; 5) acute illness or any musculoskeletal, cardiovascular or neurological disorder that could inhibit exercise or put participants at risk from exercising; and 6) unable to read and speak English. Eligible subjects will undertake a series of familiarisation sessions and baseline measurements prior to randomisation (Figure 2). The protocol has been approved (ID: 10643 CORMIE) for all participating centres by the Edith Cowan University Human Research Ethics Committee and all participants will provide written informed consent.

### Randomisation

Subjects will be randomly allocated in a ratio of 1:1:1 to the three study arms, subject to maintaining approximate balance regarding stratification for: 1) age (<60 years≥); 2) current sexual activity level (no/minor-moderate) as assessed by the European Organisation for Research and Treatment of Cancer (EORTC) prostate cancer specific questionnaire (QLQ-PR25) sexual activity score [43]; 3) previous prostatectomy (yes/no); 4) previous radiotherapy (yes/no); and 5) previous/current ADT (yes/no). A research methods consultant with no patient contact will be responsible for randomisation which will be performed in Statistical Analysis System using the Pocock-Simon minimisation algorithm.

The inclusion and exclusion criteria and stratification procedures have been designed to control for the major factors that may influence either baseline values and/or the potential for change in sexual health throughout the intervention (e.g. treatment type, time since treatment, level of sexual health both pre-treatment and at baseline, age, potential for recovery of function, incontinence). Furthermore, in addition to clinically relevant covariates, the main factors that could potentially confound the results will be assessed and adjusted for (e.g. utilisation of sexual aids [pre-treatment, post-treatment and throughout the study period], relationship status and satisfaction, any additional cancer treatment during the study period, side effects and co-morbidities influencing sexual health).

### Measurements

All measurements for primary and secondary endpoints will take place at baseline, post-intervention and 1 year follow-up. The Usual Care group will undergo an additional assessment point at the end of their delayed intervention. All groups will complete an additional assessment at 6 months follow-up involving the self-report questionnaires only. All assessments tools and procedures have established validity and reliability and are used widely in clinical research.

### Primary study endpoints

#### Sexual health

The IIEF will be utilised to assess sexual health across a variety of domains including erectile function, orgasmic function, sexual desire, intercourse satisfaction and overall satisfaction [41]. The Sexual Function scale of the EPIC is a prostate cancer specific tool which will be utilised to assess sexual function and satisfaction [42]. Sexual activity level will be assessed using the sexual domain of the QLQ-PR25 [43].

### Secondary study endpoints

Secondary endpoints include key factors associated with sexual health in men with prostate cancer.

#### Sexual self-confidence

The Short Form Psychological and Interpersonal Relationship Scale will be used to assess sexual self-confidence [44]. The Sexual Self-Efficacy Scale For Erectile Disorder will be applied to assess sexual self-efficacy [45].

#### Masculine self-esteem

The Masculine Self-Esteem Scale is designed specifically for use in men with prostate cancer and will be utilised to assess participant’s appraisal of their masculinity [46]. The Personal Attributes Questionnaire will evaluate masculinity [47].

#### Utilisation of sexual aids

A previously developed scale will be applied to assess whether participants have sought medical assistance for sexual dysfunction at any point before, during or after prostate cancer treatment [34,48]. All treatments will be documented and impact of these treatments rated.

#### Relationship satisfaction

The Dyadic Adjustment Scale will assess relationship satisfaction between participants and their partners [49].

#### Sexual supportive care needs

The level of need for help with sexual dysfunction will be assessed by the sexuality domain of the Supportive Care Needs Survey [50].

#### Quality of life

Health-related quality of life will be assessed using the Medical Outcomes Short Form 36 [51]. Quality of life will also be assessed using a prostate cancer specific tool, the EORTC QLQ-C30 and QLQ-PR25 questionnaires [52].

#### Urinary, bowel & hormonal issues

Adverse side effects specific to prostate cancer treatments will be assessed using the urinary function, bowel habits and hormonal function scales of the EPIC [42].

#### Psychological distress

The Brief Symptom Inventory-18 will be utilised to assess psychological distress across the following domains: anxiety, depression, somatisation and global distress severity [53]. Antidepressant use will also be recorded.

#### Fatigue

Cancer related fatigue will be assessed using the Functional Assessment of Chronic Illness Therapy-Fatigue questionnaire [54].

#### Body composition

Regional and whole body lean mass and fat mass will be derived from whole body dual-energy X-ray absorptiometry scans. Trunk adiposity, visceral fat and adipose indices will be assessed using standard procedures [55].

#### Body image

The Body Image Scale is a cancer-specific scale that will be used to assess participants’ perceptions of their appearance [56].

#### Physical function & physical activity levels

A series of standard tests will be used to assess physical function: 1) 400-m walk (aerobic capacity), 2) one repetition maximum in the leg press and chest press (muscular strength), 3) repeated chair rise (muscular power), 4) usual and fast pace 6-m walk (ambulation), and 5) backwards tandem 6-m walk (balance) [20,22,57]. Physical activity levels will be assessed objectively over a 7-day period using a validated, reliable tri-axial accelerometer activity monitor (ActiGraph GT3X+) [58]. Self-reported physical activity will also be assessed by the leisure score index from the Godin Leisure-Time Exercise Questionnaire [59].

#### Blood biomarkers

Blood samples will be collected and analysed commercially by accredited Australian National Association of Testing Authorities laboratories for testosterone, high sensitivity C-reactive protein (cardiovascular disease risk; also related to severity of penile vascular disease) and prostate specific antigen.

#### Exercise intervention

The exercise intervention involves a combination of aerobic and resistance exercise performed during 3 sessions per week for 6 months. The program will be supervised by accredited exercise physiologists in various exercise clinics in Western Australia, Queensland and New South Wales. The exercise sessions will be conducted in small groups of up to 10–12 participants exercising in pairs or under direct supervision to ensure correct technique and minimise the risk for injury. The exercise program is designed to provide optimal stimulus to the cardiorespiratory and neuromuscular systems while maximising safety, compliance and retention. Specifically, the aerobic exercise component will include 20 to 30 minutes of moderate to vigorous intensity cardiovascular exercise (~60-85% of estimated maximum heart rate) using a variety of modes such as walking or jogging on a treadmill, cycling or rowing on a stationary ergometer or exercising on an elliptical or cross trainer machine. Participants will be encouraged to undertake additional home-based aerobic exercise with the goal of achieving a total of at least 150 minutes of moderate intensity aerobic exercise each week. The resistance exercise component will involve 6–8 exercises that target the major upper and lower body muscle groups. Intensity will be manipulated from 6–12 repetition maximum (RM; i.e. the maximal weight that can be lifted 6 to 12 times which is equivalent to ~60-85% of 1RM) using 1–4 sets per exercise. To ensure the progressive nature of the training program, participants will be encouraged to work past the specific RM prescribed. The resistance will be increased by a 5-10% increment for the next set or training session if the subject is able to perform more repetitions than the RM specified during a set. Each session will commence with a 10-minute warm-up comprised of low-level aerobic activities as well as stretching and conclude with a 5-minute cool-down period of stretching activities. Exercise prescription will be progressive and modified according to individual response. In order to reduce the possibility of boredom and over-reaching, the exercise program will be periodised by cycling emphasis on intensity and volume. The exercise intervention has been designed in accordance with international guidelines [60,61]. Furthermore, we have used this exercise prescription effectively in previous trials involving men with prostate cancer and have reported significant improvements in libido, quality of life, lean muscle mass, fatigue, aerobic capacity, muscular strength, physical function and C-reactive protein [20,22,23,26].

#### Exercise and psychosexual intervention

Participants receiving the combined exercise and psychosexual support and self-management will complete the exercise intervention described above as well as a brief psychosexual self-management component that addresses psychological and sexual well-being. A low intensity psychological care approach will be utilised in order to maximise uptake and accessibility (i.e. facilitates translation) [62]. Specifically, at baseline, participants will receive a face to face brief psychosexual self-management session with their exercise physiologist that addresses: stress management; problem solving coping for treatment challenges; and goal setting for sexual rehabilitation. These sessions will be audiotaped with 15% reviewed to ensure adherence to the intervention protocol. The psychosexual self-management intervention will apply cognitive behavioural strategies; will utilise an adult learning approach in which men self-select goals to focus on; and will encourage self-management [63]. To support self-management men will receive a psychosexual kit that includes a published self-help book for men with prostate cancer and their partners [64]; study specific tip sheets about treatments for erectile dysfunction and goal setting for sexual rehabilitation; a progress journal-diary; and audio resources for stress management. The intervention delivered by the exercise physiologist will be manualised and based on existing materials already developed and trialled by the team [31,48]. The exercise physiologists will receive extensive training in how to deliver the intervention and continued supervision. Treatment fidelity will be managed consistent with National Institute of Health guidelines [65]. This pragmatic approach has been adopted based on the fact that men are low help-seekers and incorporating strategies that are linked to masculine ideals (i.e. exercising linked to physical strength, self-management as self-reliance, peer support offered through exercising in a group or ‘team’ of men rather than a traditional support group setting) may be more accepted by men and effective [37-39].

#### Statistical analysis

Data from our previous 3-month study in prostate cancer patients indicates that the standard deviation for change in our primary outcome of sexual health equates to ~22 points on the QLQ-PR25 sexual activity domain [26]. We observed the mean change after the intervention to differ between exercise and usual care groups by ~11 points [26]. There is no available evidence comparing changes in sexual health between exercise and psychosexual interventions. However, data from previous psychosexual interventions in prostate cancer patients indicates changes of a moderate standardised effect (*d* = 0.5) in sexual health following the psycho-educational interventions [32,33]. We have based sample size calculations on the assumption that there will be an additive effect of exercise and psychosexual therapies. A priori, 64 participants per group will be required to achieve 80% power at an alpha level of 0.05 (two tailed), and to demonstrate a difference between the three groups in sexual health at the end of the intervention. Previous experience in exercise and psychosexual therapy trials indicates an attrition rate of up to 20% over the intervention period. Therefore, to adequately ensure that we have sufficient participant numbers at the end of the intervention, 240 participants will be randomised to the study arms. A sample size of 240 will also provide us with sufficient power to detect a moderate standardised effect (*d* = 0.5) in our secondary outcomes. Based on previous experience we anticipate a further attrition rate of up to 20% over the follow-up period. Therefore, the sample size will provide 87% statistical power to detect differences between the Exercise group and the Exercise + Psychosexual group at follow-up (*d* = 0.5; n = 77 per group as the usual care group will be split between the two interventions for the prospective cohort study component of this project).

Data will be analysed using an intention-to-treat approach with maximum likelihood imputation of missing values. Analyses will include standard descriptive statistics, Student’s t-tests, chi-square, correlation and regression, and two way (group × time) repeated measures ANOVA (or ANCOVA as appropriate) to examine differences between groups over time. Clinically relevant covariates will be included in analyses. All tests will be two-tailed and an alpha level of 0.05 will be applied as the criterion for statistical significance.

## Discussion

Sexual dysfunction is one of the most prevalent, distressing and lasting consequence of prostate cancer treatment [2-11]. Despite this, very little is known about the management of sexual dysfunction which is reflected by the fact that current health care services do not adequately meet the sexual health needs of prostate cancer survivors [16]. Consequently, a significant proportion of prostate cancer survivors have profoundly reduced quality of life and struggle to come to terms with redefining their identity as a man [2-4,10,12-14,66,67]. Clearly, the management of sexual dysfunction is a critical survivorship care issue. This project will directly improve cancer outcomes by enhancing survivorship care through the development of effective, evidence-based supportive care services. This project will be the first comprehensive examination of the potential role of exercise in the management of sexual dysfunction in prostate cancer survivors. Innovation is further exemplified by proposing and evaluating a potential best-practice management approach, integrating pharmacological (usual medical care), physiological (exercise program) and psychological (brief psychosexual self-management) treatment modalities to address the complex and multifaceted aetiology of sexual dysfunction following cancer. Furthermore, the comprehensive evaluation of factors associated with sexual health will allow for a detailed analysis of the mechanisms driving change in sexual dysfunction following exercise and psychosexual therapies. We expect dissemination of the knowledge gained from this study to advance cancer care by improving sexual health, masculine self-esteem, quality of life, psychological distress, fatigue, body composition, physical function and physical activity levels as well as reducing risk factors for co-morbid conditions. Knowledge gained will help expand clinical guidelines for the effective management of sexual dysfunction secondary to prostate cancer and may lead to a paradigm shift in current practice. The ultimate outcome will be improved care for prostate cancer survivors through enhanced supportive care practice and policy.

## Abbreviations

ADT: Androgen deprivation therapy; RCT: Randomised controlled trial; IIEF: International index of erectile functioning; EPIC: Expanded prostate cancer index composite; EORTC: European Organisation for Research and Treatment of Cancer; QLQ-PR25: European Organisation for Research and Treatment of Cancer prostate cancer specific quality of life questionnaire; QLQ-C30: European Organisation for Research and Treatment of Cancer quality of life questionnaire for people with cancer; RM: Repetition maximum; 1RM: One repetition maximum.

## Competing interests

The authors declare that they have no competing interests.

## Authors’ contributions

PC, SKC, RUN, NS and DAG developed the study concept and protocols and initiated the project. RAG, DRT, DJ and MAH assisted in further development of the protocol. RAG, NS, DJ, MAH, PChong and DH will provide access to patients. PC, SKC, RUN, DRT, KH and DAG will implement the protocol and oversee collection of the data. PC drafted the manuscript, all authors contributed to revisions and all authors approved the final manuscript.

## Pre-publication history

The pre-publication history for this paper can be accessed here:

http://www.biomedcentral.com/1471-2407/14/199/prepub
